# Separation of Oligosaccharides from Lotus Seeds via Medium-pressure Liquid Chromatography Coupled with ELSD and DAD

**DOI:** 10.1038/srep44174

**Published:** 2017-03-09

**Authors:** Xu Lu, Zhichang Zheng, Song Miao, Huang Li, Zebin Guo, Yi Zhang, Yafeng Zheng, Baodong Zheng, Jianbo Xiao

**Affiliations:** 1 College of Food Science, Fujian Agriculture and Forestry University, Fuzhou 350002, China; 2 Teagasc Food Research Centre, Food Chemistry and Technology Department, Moorepark, Fermoy, Co.Cork, Ireland; 3 Institute of Food Science and Technology, Fujian Agriculture and Forestry University, Fuzhou 350002, China; 4 Institute of Chinese Medical Sciences, State Key Laboratory of Quality Research in Chinese Medicine, University of Macau, Taipa, Macau

## Abstract

Lotus seeds were identified by the Ministry of Public Health of China as both food and medicine. One general function of lotus seeds is to improve intestinal health. However, to date, studies evaluating the relationship between bioactive compounds in lotus seeds and the physiological activity of the intestine are limited. In the present study, by using medium pressure liquid chromatography coupled with evaporative light-scattering detector and diode-array detector, five oligosaccharides were isolated and their structures were further characterized by electrospray ionization-mass spectrometry and gas chromatography-mass spectrometry. *In vitro* testing determined that LOS3-1 and LOS4 elicited relatively good proliferative effects on *Lactobacillus delbrueckii* subsp. *bulgaricus.* These results indicated a structure-function relationship between the physiological activity of oligosaccharides in lotus seeds and the number of probiotics applied, thus providing room for improvement of this particular feature. Intestinal probiotics may potentially become a new effective drug target for the regulation of immunity.

Lotus seeds are mature seeds of *Nelumbo nucifera* Gaertn and are so far the oldest plant seeds currently known. In China, lotus seeds have been traditionally used as both pharmaceutical and food resource. Bioactive ingredients such as water-soluble carbohydrates, alkaloids, flavonoids, and superoxide dismutase (SOD) render various bioactivities to lotus seeds that regulate intestinal and stomach functions, prevent oxidation, lower blood glucose, and boost immunity. Thus, lotus seeds are widely used in southeastern Asia and America as a dietary supplement for daily health care^1^,^2^. Oligosaccharides in lotus seeds also enhanced the tolerance of *Bifidobacterium adolescentis* to acid, bile, and digested fermented food in a simulated gastrointestinal environment, improved the levels of acetic acid, propionic acid, and butyric acid that are produced by *B. adolescentis*, and enriched the probiotic potential of gastrointestinal microorganisms. The main oligosaccharides in lotus seeds are disaccharides, trisaccharides, and tetrose, which contain glucosidic bonds such as Manp-(1→, Galp-(1→, α(1→6)-Glup and α(1→6)-Manp^3^,^4^. However, the specific structure of each saccharide component remains unknown, thus requiring qualitative and quantitative analyses, which may particularly help in the fields of medicine, agriculture, and functional food industry. Furthermore, research studies on the large-scale fabrication of oligosaccharides are warranted.

Column chromatography is commonly used in the synthesis of pharmaceuticals and in the large-scale separation and purification of bioactive natural products because of its low cost and ease in operation. However, this method has drawbacks including complex operations and the use of massive amounts of organic solvents. Furthermore, when assessing carbohydrates that do not adsorb ultraviolet light, a phenol-sulfuric acid approach is often applied for its offline detection, which in turn further prolongs collection time, utilizes a higher number of samples, as well as imposes health hazards to analysts. Thus, flash chromatography has been developed, which provides advantages such as high resolution, a high automatic level (including autosampler and peak collection), and program control of the gradient. It effectively overcomes the abovementioned issues, and based on the applied force, flash chromatography can be divided into two types: rapid chromatography (0.1–5/10 bars) or medium-pressure liquid chromatography (MPLC, 5/10–50 bars)^5^. Compared to similar techniques such as high-efficiency liquid chromatography and high-speed counter-current chromatography, the amount of products prepared via flash chromatography can be relatively high, with samples ranging in weight from milligrams to hectograms, which are features that play an important role in the separation and purification of natural products^6^. With the coupling of a refractive index detector (RID), flash chromatography may further be used in the separation of plant functional low-molecular weight carbohydrates^7^,^8^. Currently, the application and research studies on oligosaccharides in a fully automated rapid chromatographic system are rarely reported.

Some of the oligosaccharides with similar degrees of polymerization showed high polarity and low thermal stability; therefore, it is a challenge to separate oligosaccharides with similar polarity. Currently, when separating and purifying a large amount of oligosaccharides, activated carbon chromatography, anion-exchange chromatography (AEC), and seize-exclusion chromatography (SEC) are often used. However, the single use of these technologies still encounters issues such as low sample resolution and difficulty in removing impurities. Thus, AEC and SEC technologies have been combined to achieve better results^9^, although the steps are relatively complicated and its efficiency may be low. Moreover, because gradient elution is frequently needed when separating a large amount of oligosaccharides using the AEC technique, RID or ELSD detectors thus cannot be used, and only manual collection is possible, thereby limiting the application of this technique. Hydrophilic interaction liquid chromatography (HILIC) shows good retention and separation selectivity of strongly polar compounds and hydrophilic compounds, and is a highly orthogonal method that is complementary to reversed-phase liquid chromatography. After modifying the polarity of solvents, HILIC shows both normal and reversed phase separation models, thereby demonstrating that HILIC may be utilized for one-time separation of bioactive phytochemicals. In the late 1990s, high-efficiency liquid analytical chromatography was applied for the analysis of different types of carbohydrates. Furthermore, its coupling with flash chromatography has yielded superior fabrication and purification results.

This study innovatively used the techniques of medium-pressure liquid chromatography coupled with ELSD and DAD to separate and prepare large amount of micromolecular carbohydrates including micromolecular carbohydrate isomerizes from lotus seeds to further analyze the structure of oligosaccharides. Unlike traditional methods, the method developed in this study does not require complicated pre-treatment processing, such as depigmentation and extraction, thereby facilitating in large-scale isolation using two steps within a short period of time. The present study has developed a high-resolution, automatic, and reproducible method for the rapid purification, separation, and fabrication of oligosaccharides from lotus seeds (Fig. 1). The techniques of cation exchange chromatography, Q-TOF-MS, and GC-MS are also used for the determination of purity and structural features, as well as to establish the structure-function relationship between lotus seed oligosaccharides and the proliferation of the bacterium *Lactobacillus bulgaricus*; therefore, it provides new ideas and methods for fabricating a large amount of highly purified oligosaccharides from natural medicinal plants that possess prebiotic activity.

## Results and Discussion

### Optimization of MPLC

One major characteristic of the stationary phase detected by hydrophilic column chromatography is that strongly polar groups that have superior affinity towards water are found on the surface of the stationary phase; for example, the stationary phase may be an amino group, a hydroxyl group, or an amino group. In particular, the amino group shows great separation selectivity towards strongly polar compounds such as saccharides, organic acid, and nucleosides^10^, and most existing chromatographic column manufacturers provide an amino-bonded phase for HILIC. It takes a relatively long balance time for an amino functional silicone emulsion to colloid^11^; at the same time, the properties of stationary silicone emulsion phase of an amino column are relatively active. Thus, when in use, an irreversible adsorption phenomenon may easily occur^12^, thereby resulting in the deterioration of spectrum peaks^13^. Compared to an amino group, an amide group has relatively low activity, and its alkalinity is also relatively weak. The retention of materials in an amide bonded phase is rarely influenced by pH, thus ligands do not easily run off, and a relatively high recovery rate can be obtained^14^.

**Figure 2:**
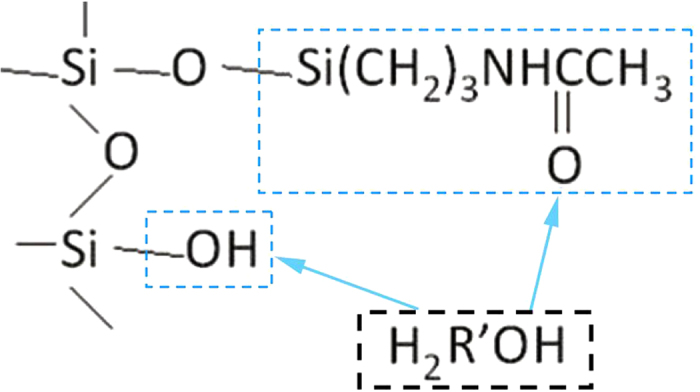
A diagram illustrating the interaction of compounds in the stationary phase and mobile phase in the amide-type hydrophilic chromatographic column.

The chromatographic column of the present study employed an aminopropyl-bonded silica gel as the packing material. When preparing this packing material, TEOS are used to treat the surface of silica gel, reduce the number of isolated silicon hydroxyl on the surface, and lower the activity of silicon hydroxyl on the surface of the silica gel, thus preventing the over-adsorption of polar compounds. In the reversed-phase system, the material of this chromatographic column has opposite retention characteristics compared to the C_18_ material, whereas in the positive phase system, its retention force is relatively weaker compared to NH_2_. Thus, the system is able to elute materials that are generally difficult to elute using the positive phase system or retain very weak materials in the reversed phase system. This material also has relatively good stability in the water-containing flow phase, thus compensating the drawbacks of the traditional NH_2_ saccharide column^15^ by facilitating a quick and high efficiency separation of strongly polar samples. The operational process is illustrated in Fig. 2.

### The influence of flow rate on the separation efficency

Lotus seeds contain a large number of alkaloids such as dauricine, lotusine, nuciferine, pronuciferine, liensinine, isoliensinine, roemerine, neferine, and armepavine. Procyanidins were also isolated from the lotus seed pod. Seeds also contain gallic acid, isoquininolinol, saponins, and carbohydrates^1^,^16^.

The bioactive components in lotus seeds may be extracted by using the hot water extraction method. After the liquid extract is sequentially treated with a high concentration ethanol, centrifugation, rotary evaporation, strong polar materials such as most macromolecular polysaccharides, proteins, and polypeptides, and weakly polar materials such as volatile terpene and lipid are removed. The extract is acidic, thus benefitting the retention of highly polar aqueous matters such as low-molecular carbohydrates, alkaloids, polyphenols, flavonoids, and amino acids. At initial separation, the ratio of acetonitrile in the mobile phase is relatively high, and the HILIC displays a relatively strong positive phase chromatographic separation mode. Lotus seeds are extracted using an aqueous phase; therefore, the content of non-polar materials in the solute is relatively low, and the positive phase separation mode works well under this condition. For HILIC chromatography, during the process that the ratio of water in the mobile phase increases from 0% to 100%, due to the existence of hybrid mechanism, the retention of solutes displays a “U” shape. When the organic solvent and water are mixed as the mobile phase, complex forces such as hydrogen bonds and dipole moments exist between molecules. When the ratio of water in the mobile phase increases from 0 to 50% (v/v), the chromatographic column shows typical characteristics of HILIC retention, and water becomes a strong elution agent. When the ratio of water further increases to 50%, the retention of solute increases and water thus becomes a weak elution agent, and solute retention satisfies the reverse phase chromatographic retention mechanism, which is not beneficial for the retention of glycosides. Based on the experimental results, to shorten the experiment duration, the ratio of water as the solute of the mobile phase should be within 15–35%.

**Figure 3:**
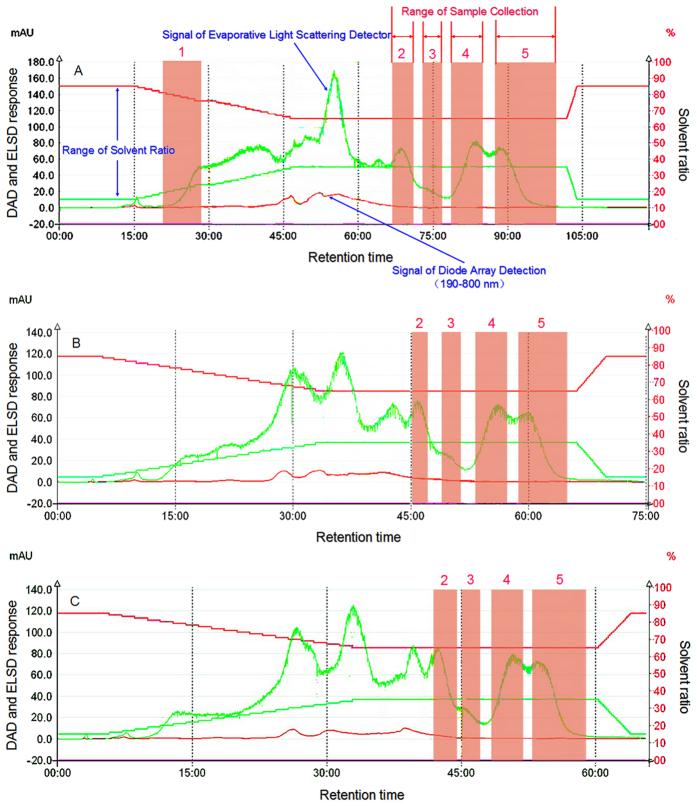
The partition chromatogram of eacholigosaccharide component in lotus seeds with different flow rates (the sample concentration is 0.2 g/mL, the acetonitrile concentration is 85–65%, the variation rate is 2/3%/min), (**A**) 30 mL/min; (**B**) 45 mL/min; (**C**) 60 mL/min.

Figure 3A–C show that although the increase in flow rate decreases the time required for separation, when the low rate was 30 mL/min, the separation time was relatively long. When the flow rate was increased to 45 mL/min, the time needed for separation decreased. When the flow rate was 60 mL/min, the separation time of oligosaccharides from lotus seeds was significantly shortened, resulting in a partial mixture of compound 2 with some material showing UV adsorption peaks, which in turn separated products that were of lower purity. The relatively large flow rate reduced the allocation times of solutes and stationary phase in the extract. That is, the interaction time between the solute and the stationary phase was markedly shortened, thereby reducing the retention time. Meanwhile, when superimposition degree among peaks 2–5 increased, the separation degree decreased. Although the separation time period was shortened, the overall solvent assumption amount was large (3,600 mL), and thus it would be ideal to select 30 mL/min as the separation flow rate.

**Figure 4:**
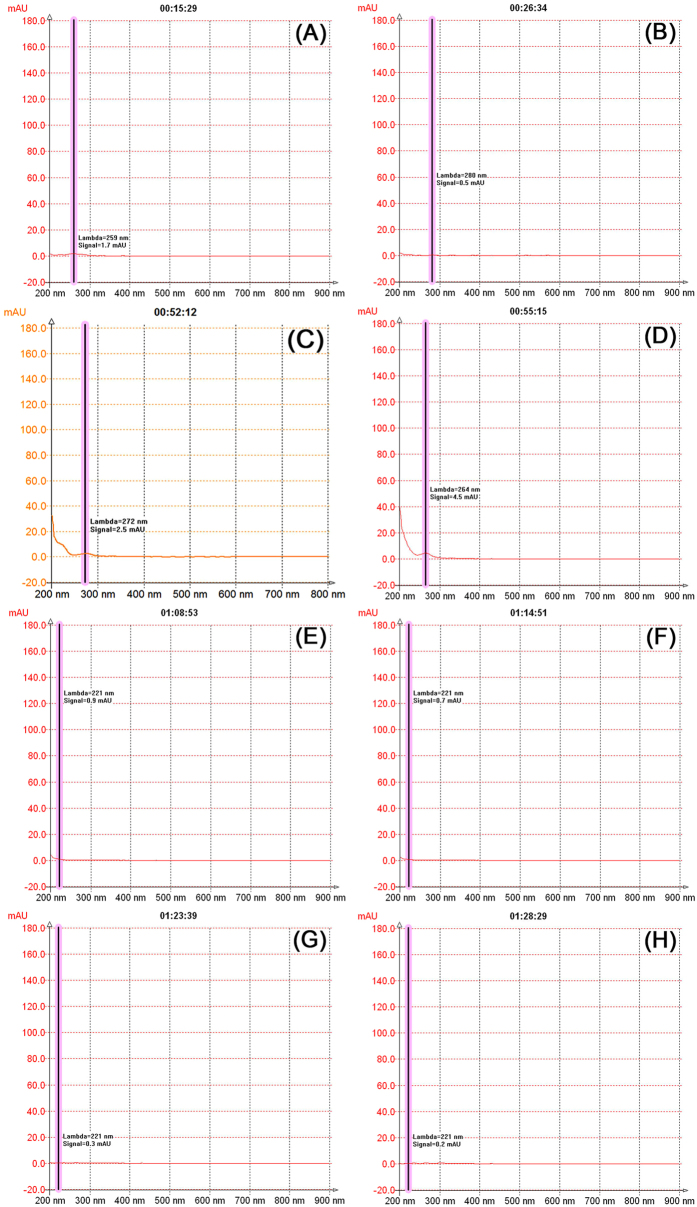
The DAD spectrum of the solution at a flow rate of 30 mL/min at a specific time.

Figure 4A shows that when the separation time is around 15 min, the maximum UV adsorption wavelength of the solution is 259 nm, which corresponds to a solute that produces the first peak whose wavelength is similar to the maximum adsorption wavelength of lipid materials^17^. This solute shows the strongest non-polarity in lotus seeds. After 20–40 min, weakly polar materials in lotus seeds such as saturated alcohols, aldehydes, esters, and acids that contain no UV adsorption (Fig. 4B) start to outflow from the chromatographic column. With the increase of water in the mobile phase, the separation mode becomes a weakly positive chromatographic mode, and the retention ability of polar materials in the stationary phase decreases. Within the time period of 40–68 min, a relatively strong UV adsorption peak was observed, indicating that the relatively strongly polar materials have started to display peaks. During this period of time, the solution constantly shows an adsorption peak at 190 nm, which is mainly due to the strong adsorption of UV lights by peptide bonds at 190 nm. This adsorption peak might be generated by materials such as micro-molecule polypeptides or amino acids in lotus seeds. At around 52 min (Fig. 4C), the maximum adsorption peaks of the UV spectrum were around 215 nm and 280 nm. At around 55 min (Fig. 4D), the maximum adsorption peak was 265 nm, indicating that at this time, the primary compounds were alkaloids, gallate, saponins, and alcohol-soluble proteins^18^,^19^,^20^,^21^,^22^. The UV adsorption peak of polyphenol material was not detected, which may be related to the lowered solubility of polyphenol because the aqueous extract of lotus seeds was acidic. Figure 4E–H show that except for the area collected by peak 2 that introduces some materials containing UV adsorption groups, peaks 3–5 did not show introduction of this material, indicating that peaks 2–5 (red area in the figures) may represent carbohydrates. The shape of the peak indicates that strongly non-polar and weakly polar materials are hard to separate due to their simple content and various types; however, because the retention time of each strongly polar material is relatively long, these can be preliminarily separated.

### Influence of sample concentration on separation efficiency

**Figure 5:**
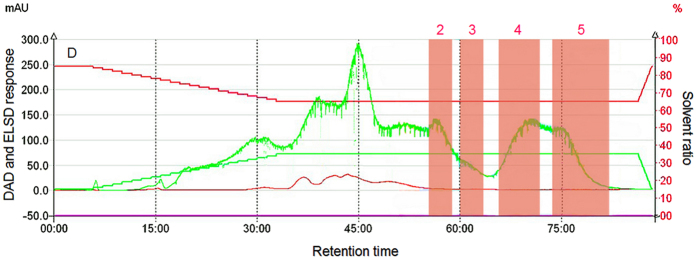
Partition chromatographic diagram of each oligosaccharide component in lotus seeds under different sample concentration conditions (the sample concentration is 0.4 g/mL, the acetonitrile concentration is 85–65%, the flow rate is 30 mL/min).

Comparison of Figs 3 and 5 shows that when the flow rate is fixed (30 mL/min) and the sample concentration of the sample is doubled, although peaks 2–4 show no introduction of saccharide, the separation degree of each compound was relatively poor, and the shape of the peak corresponding to each compound had markedly changed, thereby indicating a decrease in the separation effect. This may be related to the overloading of samples and the existence of inorganic salts in the extract of the lotus seed. The overloading of sample may reduce the reaction points between stationary phases of the solutes. Moreover, ions generated by the ionization of inorganic salts in the extract may easily interact with water molecules in the mobile phase, thereby producing static electricity or hydrogen bonds, ultimately resulting in the weakening of capacity of strong elution agents to react with the solutes. With a higher salt concentration, the reduction in the elution ability of water was more significant, which was indicated by the shortening in the retention time of saccharide solutes. Solutes such as polyphenol^23^ in the extract of lotus seeds that is acidic after ionization may show an electrostatic repulsive reaction with the remaining silicon hydroxyl after dissociation. With the increase in salt concentration in the mobile phase, the electrostatic force applied to the polyphenol materials continued to increase. The carbohydrate compounds are weak electrolytes, which receive a smaller electrostatic effect compared to phenol materials. The retention process showed a mixed mode of HILIC and ion exchange. Thus, the increase in the sample concentration may result in the supposition of the UV adsorption peak of the solutes and the peak of the carbohydrate solutes. Accordingly, 0.2 g/mL was selected as the sample concentration of oligosaccharides extracted from lotus seeds.

### The influence of gradient elution on the separation efficiency

**Figure 6:**
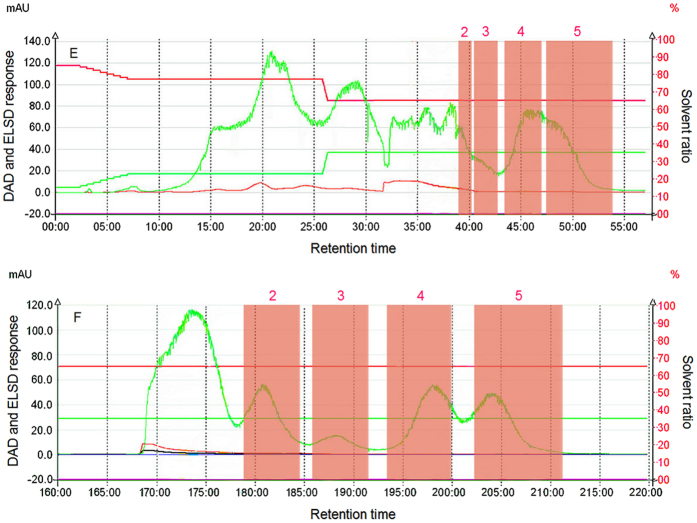
Partition chromatographic diagram of each oligosaccharide component in lotus seeds under different acetonitrile concentration conditions (the sample concentration is 0.2 g/mL, the acetonitrile concentration is 85–65%, the flow rate is 30 mL/min, the ratio of acetonitrile is 65%).

The separation process was modified, in which the ratio of acetonitrile was decreased from an initial value of 85% (2.33 min) to 77% (7.33 min) (rate of decrease: 1.6%/min). Later, the ratio of acetonitrile remained the same for 18 min, and then decreased from 77% (25.33 min) to 65% (26.33 min) until the end of the separation, with a decrease rate of 2.4%/min. Figure 6E shows that before the appearance of peak 2, a very small portion of the material corresponding to the previous peak was non-polar, as indicated by the time of peak appearance not varying with the ratio of mobile phase. After separation, the material corresponding to peak 2 showed no introduction of non-carbohydrate impurities, and the separation of peaks 3–5 was very poor, indicating that the rapid decrease in the acetonitrile ratio leads to better a interaction between solutes and aqueous phases of the solvent, which is not beneficial for the separation of oligosaccharide in lotus seeds. Under a typical HILIC mode, the mobile phase requires a strongly polar system, and often, a mobile phase containing water is used to provide an essential elution force. When a gradient elution is needed, often the initial mobile phase has high organic matter, and the content of aqueous phase gradually increases, which is consistent with the rules of gradient elution of positive phase chromatography.

In the second equal variation process, the ratio of acetonitrile in the mobile phase was decreased from an initial value of 85% (which remained for 145 min) to 65% (after 160 min), which was remained until the end of the separation. Figure 6F shows that most materials before the appearance of peak 2 were non-polar or weakly polar. Comparison of Figs 3, 4 and 6F shows that because the ratio of 85% of the acetonitrile remained for 140 min, no peak for the solute was observed, indicating that the polarity of the aqueous solute in lotus seeds was much higher than that in the 85% acetonitrile, and the dissolution of the aqueous phase in the 85% acetonitrile was relatively low, and thus the aqueous phase remained in the stationary phase of the chromatographic column. To separate material, gradient elution or other methods were required. Later, a step that rapidly lowered the content of acetonitrile allowed all solutes that had adsorbed in the stationary phase to be eluted. The chromatography changes from a positive phase separation mode to a weakly positive phase mode, the retention time of the weakly polar matters in the stationary phase is relatively short, and the retention time of the relatively strongly polar matters in the stationary phase were all relatively long. Acetonitrile is an aprotic solvent, which is suitable to separate carbohydrates. When aqueous organic solvents were used as mobile phases, a “water-concentrated layer” appeared on the surface of the hydrophilic stationary phase, and the retention of four compounds after peak occurrence was realized by the allocation effect of the “water-concentrated layer” and the mobile phase^24^. After isocratic elution using a low acetonitrile ratio, the four compounds occurred in the chromatographic column as high-retention factors for a long time, the allocation effect increased, and thus the separation degree was relatively high. Overall, the separation degrees of weakly non-polar and weakly polar matters were extremely low, and the polarity of peaks 2–5 for strongly polar matters increased sequentially, indicating a positive phase chromatographic mode. The highly polar compounds collected by this method and corresponding to the four peaks were well separated and contain relatively few impurities, demonstrating good separation effects. For poorly polar materials, a gradient and slow method using acetonitrile was used to collect these materials. In conclusion, to ensure the purification of compounds, the material corresponding to peak 1 in Fig. 3A, and the materials corresponding to the peaks 2–5 in Fig. 6F were used in the secondary separation and purification using an optimized process.

### Secondary separation of collected compounds

**Figure 7:**
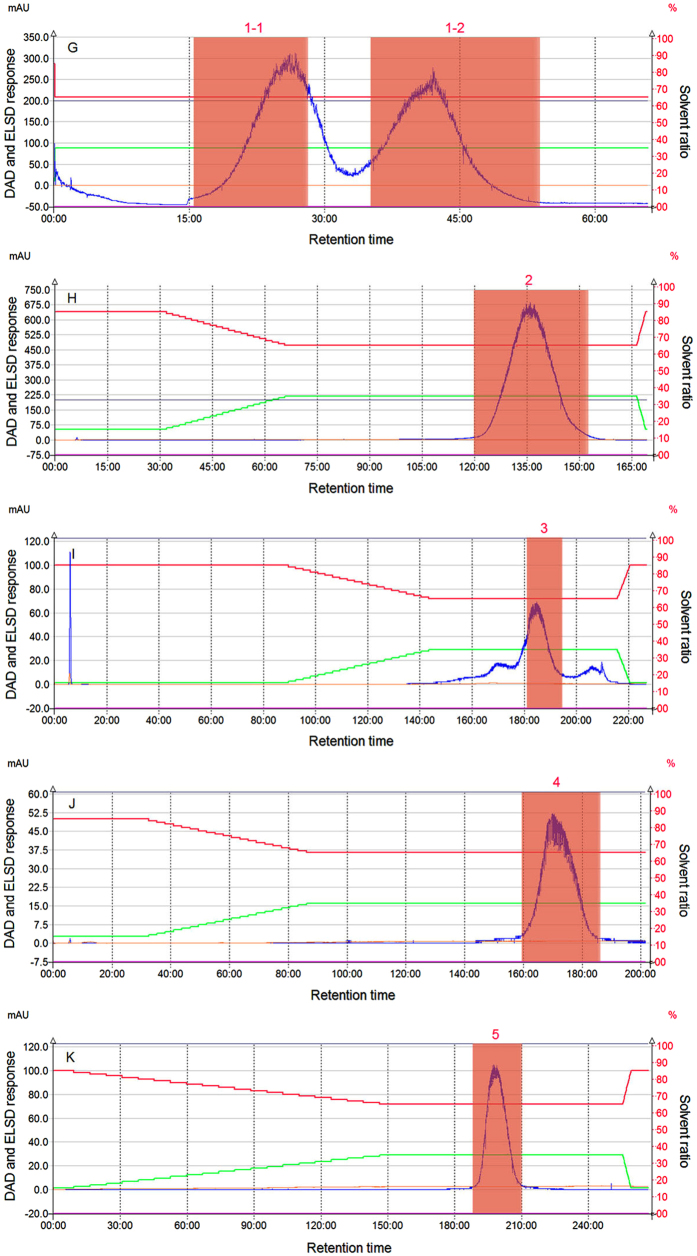
A secondary partition chromatographic diagram of each collected oligosaccharide component in lotus seeds (the acetonitrile concentration is 85–65%, and the flow rate is 30 mL/min).

A slow variation rate in the content of acetonitrile enhanced the separation of non-polar and weakly polar materials, and after the secondary separation, the materials corresponding to peak 1 in Fig. 7G were separated into two components. Because peaks 1–2 were asymmetric, its component stayed as a mixture. The materials in the red area of peak 1-1, as well as peaks 2–5 in Fig. 7H–K were thereby named as LOS1, LOS2, LOS3-1, LOS3-2, and LOS4, respectively, after being freezed and dried to be powders for further purification and structural analysis.

### Analysis of contents of oligosaccharides in lotus seeds

**Figure 8:**
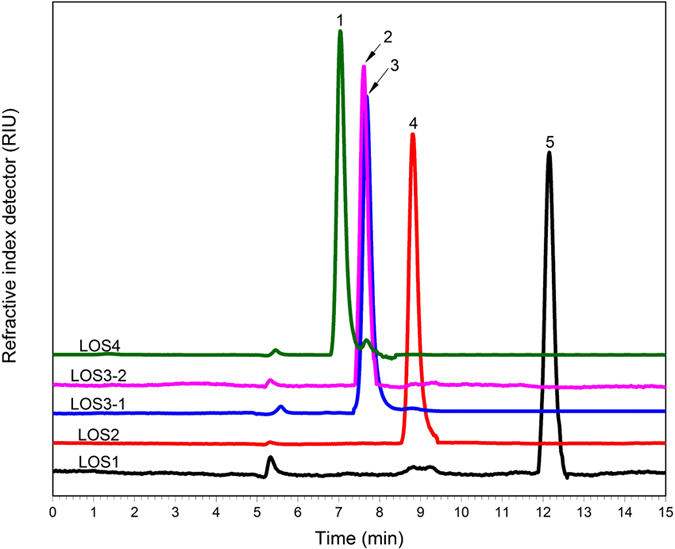
The HPLC chromatographic spectrum of each oligosaccharide component in lotus seeds.

The purification degree of the obtained components and recycling rate were analyzed using HPLC-ELSD, and the cation exchange chromatographic method was used for separation based on the interaction between hydroxyl of oligosaccharides and metallic ions in the cation exchange groups that have sulfonic acid group. Different carbohydrates with various structures are often separated based on the polymerization degree of carbohydrates. Compared to Fig. 8, for the five compounds LSO4, LSO3-2, LSO3-1, LSO2, and LSO1 separated from aqueous oligosaccharide extract in lotus seeds, the separation degree of each compound was good; the chromatographic peak was distinct, sharp, smooth, and symmetric. The retention time was 7.032, 7.621, 7.689, 8.799, and 12.149 min, respectively, which is just opposite to the peak occurrence order in HILIC. These observations indicate that the major cation exchange force was that of hydroxyl charge repulsion, whose purification degrees were 96.76%, 98.07%, 98.18%, 99.74%, and 92.67%, respectively. After extraction and separation, each compound in 100 g of lotus seeds were of high purity, showing a weight of 1.107 g, 0.554 g, 0.183 g, 0.443 g, and 0.243 g. A 1,000-mg frozen and dried sample was used after ethanol elution to remove macromolecular starches, proteins, and polysaccharides. After an optimization process and secondary separation, highly purified compounds with a weight of 186.283 mg, 93.170 mg, 30.296 mg, 72.924 mg, and 42.672 mg may be obtained, respectively. By liquid chromatography, a large amount of active compounds from lotus seeds could be effectively concentrated and separated within a short time.

### Analytical results of the Q-TOF-MS spectrum

**Figure 9:**
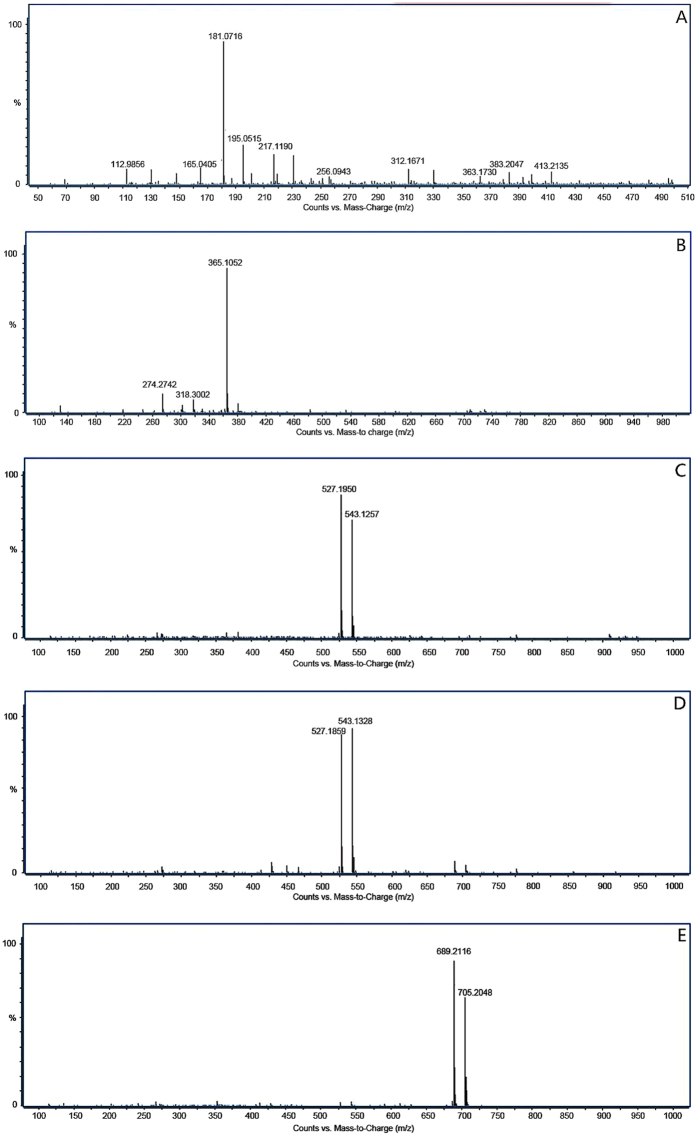
The Q-TOF-MS analytical results of oligosaccharide compound 1, 2, 3, 4, and 5 in lotus seeds.

The oligosaccharide extract from lotus seeds primarily contained compounds 1, 2, 3–1, and 4, corresponding to a first-order mass spectrum Fig. 9(A–F), respectively. Unlike proteins that are easily protonized, carbohydrates may easily form adducts with Na^+^ and K^+^ in the samples or matrix. Only a few ion peaks were observed for each compound, thereby indicating a high purification degree. Data were analyzed using Mass Hunter Workstation Software (version B.04.00 Qualitative Analysis, Agilent Technologies, Santa Clara, CA, USA), and the matching degrees between the molecular formula and the corresponding molecular formula are listed in Table 1. From above results, the formula weight of oligosaccharide compounds 1, 2, 3–1, 3–2, and 4 in lotus seeds as calculated by the software were consistent with their theoretical molecular formula^25^. The molecular weight of chemical 1 was 182, which is consistent with that of sugar alcohols. Compound 1 did not display colors in the thin-layer chromatography, thereby indicating that it is not a carbohydrate compound, and its specific structure needs further investigation. The DP of compounds 2, 3, 4, and 5 were 2, 3, 3, and 4, respectively, and the molecular weight difference between oligosaccharides with different polymerization degrees was 162, corresponding to monosaccharide residue breakage from a reducing end or non-reducing end. This indicates that there should be no acidic groups with relatively large molecular weights such as carboxylic acid and sulfuric acid in all the oligosaccharides extracted from lotus seeds. Further, the ionization method and molecular weight of the examined oligosaccharide structures were not consistent with that of alkaline oligosaccharides, indicating that there was no amino group in each compound structure. The oligosaccharides in lotus seeds can be effectively detected using a positive ion mode, whereas in the negative ion mode, the abundance of fragments was relatively low, indicating that the oligosaccharides possess weak electrolyte properties of neutral carbohydrates^26^, and thus each oligosaccharide compound is a neutral oligosaccharide. The above results indicate that LOS3-1 and LOS3-2 are isomers, demonstrating that the HILIC mode can identify a trivial difference in the force between an isomer of the target matter and the stationary phase induced by spatial isomerism. The polarity and polymerization degrees of compounds 1, 2, 3–1, 3–2, and 4 were arranged in an order from weak to strong, indicating that the polarity of these compounds are relatively strong, and the existence of polar functional groups in the stationary phases in the hydrophilic chromatographic column effectively prolonged the retention time of the previous portion of polar compounds. Furthermore, the chromatographic condition after optimization may solve the separation issue between non-polar natural products and strongly polar carbohydrates in lotus seeds. As a mobile phase, a medium polar solvent suits the retention and separation behavior of oligosaccharides from lotus seeds in HILIC.

### Methylation analysis

**Figure 10:**
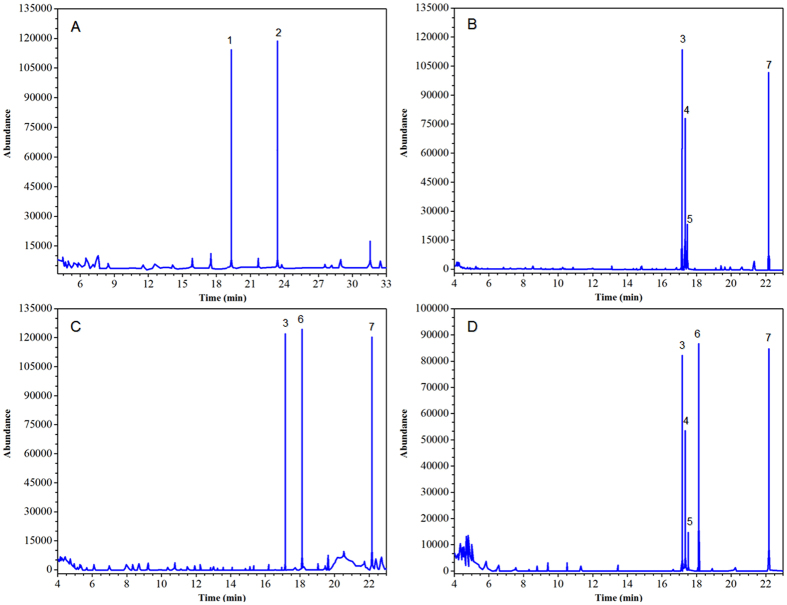
GC–MS profiles of methylated lotus seeds oligosaccharides (**A**) LOS2; (**B**) LOS3-1; (**C**) LOS3-2; (**D**) LOS4.

Methylation analysis was used to analyze each compound monomer in the oligosaccharides extracted from lotus seeds (Fig. 10). Table 2 summarizes the types of alditol acetate products after methylation of each purified oligosaccharide compound, and the fraction method of ion fractions measured using GC-MS. The types of glyosidic bond in each compound and the corresponding peak occurrence time are listed in Table 3. The methylation products of LOS2 primarily consisted of a non-reducing t-Gal*p* residue (1,5-Ac_2_-2,3,4,6-Me_4_-galactitol), and terminal t-Glc*p* residue (1,4,5-Ac_3_-2,3,6-Me_3_-glucitol), where the molar ratio was approximately 1:1, and their structures were similar to the structure of lactose. The methylation products of LOS3-1 primarily consisted of a non-reducing t-Man*p* residue (1,5-Ac_2_-2,3,4,6-Me_4_-mannitol), terminal t-Fru*f* residue (2,5-An_2_-1,3,4,6-Me_4_-mannitol and 2,5-An_2_-1,3,4,6-Me_4_-glucitol), and t-Glc*p* residue (1,5,6- Ac_3_-2,3,4-Me_3_-glucitol), where the molar ratio was approximately 1:1:1. In these structure, terminal t-Fruf residue produced two types of epimer derivatives: mannitol and glucitol^27^,^28^,^29^, indicating that the fructose group was at the end of LOS3-1. The methylation products of LOS3-2 primarily consisted of a non-reducing t-Man*p* residue (1,5-Ac_2_-2,3,4,6-Me_4_-mannitol), t-Man*p* residue (1,5,6-Ac_3_-2,3,4-Me_3_-mannitol), and t-Glc*p* residue (1,5,6-Ac_3_-2,3,4-Me_3_-glucitol), where the molar ratio was approximately 1:1:1. The methylation products of LOS4 primarily consisted of a terminal t-Fru*f* residue, non-reducing t-Man*p* residue, t-Glc*p* residue, and t-Man*p* residue in the methylation products of LOS3-1 and LOS3-2, where the molar ratio was approximately 1:1:1:1. In lotus seeds, a monosaccharide among dissolvable macro-molecular polysaccharides may refer to galactose, fructose, seminose, and glucose^1^, and the results of methylation indicated that LOS2 is lactose, and the structures of LOS3-1, LOS3-2, and LOS4 were highly similar to each other. The four micro-molecular carbohydrates all showed straight chain structures and the same monosaccharide component, indicating that the polysaccharide compounds in lotus seeds are assembled and synthesized during the growth of the lotus plant. In addition, LOS3-1 and LOS3-2 may be precursors of LOS4. The methylation results were also consistent with the polymerization degree of each carbohydrate obtained from the analysis of Q-TOF-MS. At the end of LOS3-1, furanose was detected, and LOS3-1 showed an earlier peak occurrence than LOS3-2 in HILIC, indicating that the spatial hindrance formed by furanose at the end of the structure that intersects pyranose is smaller, and the polarity of the carbohydrate compound in LOS3-1 is weaker than that of LOS3-2.

### The influence of each carbohydrate compound separated from lotus seeds on *L. delbrueckii* subsp. bulgaricus, short-chain aliphatic acid, and lactic acid

Bulgaria is the home to yoghourt, and in 1905, the Bulgarian scientists Stamen Grigorov separated bioorganisms from the yoghourt to identify the existence of lactic acid bacteria (LAB), thus the denomination of *L. delbrueckii* ssp. *bulgaricus* in LAB is related to the nationality of the scientist who discovered this. During the fermentation process, the lactobacilli will release certain dissolvable factors and metabolites such as proteins, cell wall constituents, lactic acid, and DNA. LAB somatic cells and these dissolvable factors may, via a toll-like-receptor, recognize activated dendritic cells, macrophages, or monocytes. In addition, to some extent, a specific helper T(Th) reaction may be triggered, which activates the APCs in the gastrointestinal tract, thus further influencing the general immune response and inducing an immune regulatory effect^30^,^31^. Although most *Bifidobacterium* strains have been proven to easily utilize oligosaccharides, only a few bacterial strains in lactobacillus, such as *L. delbrueckii* ssp. *Bulgaricus*, show this ability. To date, utilizable oligosaccharides that have been discovered so far include fructo-oligoses (FOS) and galatooligosaccharides (GOS)^32^.

**Figure 11:**
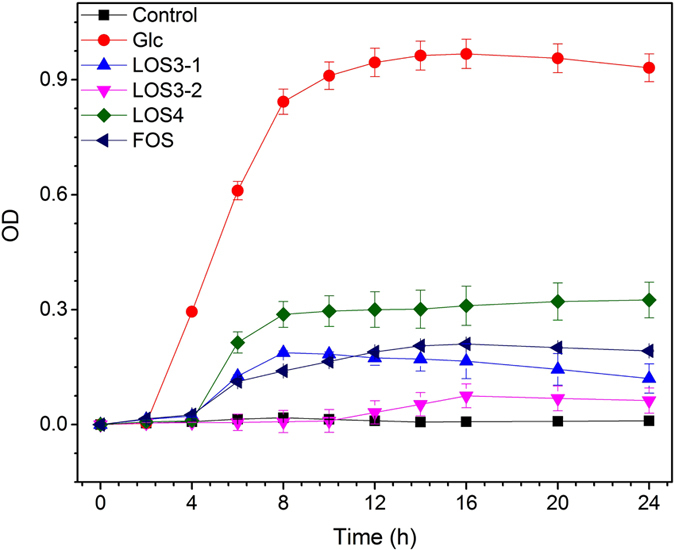
Growth curves of *L. delbrueckii* ssp. *Bulgaricus* incubated for 24 h in media containing Glc, LOS3-1, LOS3-2, LOS4, FOS. *Error bars represent the standard deviation of replicates (*n* = 3).

Figure 11 indicates that *L. delbrueckii* ssp. *bulgaricus* grows best in substrates that use Glc as its carbon source. After 24 h, the thallus density was significantly higher in substrates where Glc was the carbon source than in substrates where LOS3-1, LOS3-2, LOS4, or FOS was the carbon source. After *L. delbrueckii* ssp. *bulgaricus* was cultivated in the substrates where LOS3-1, LOS4, or FOS was the carbon source for 6 h, the number of thalli and the growth rate showed no significant increase (*p* > 0.05). In substrates where LOS4 was the carbon source, the number of thalli showed a higher growing rate in the logarithmic phase than that in substrates where LOS3-1, LOS3-2, or FOS was the carbon source, and its propagation effect was superior among the oligosaccharides. The three major systems that utilized *L. delbrueckii* ssp. *bulgaricus* were as follows: (1) those that consist of a proton transport system (PTS) and glycosidase system; (2) those in which permease is integrated with an oligosaccharide substrate outside the cell membrane, and under the condition of energy supply, the reversed difference in concentration transports the material out of the thallus; and (3) those in which during metabolism, the transmembrane protein of the one-way substrate transporter in the thallus that has ATP-binding sites that transport oligosaccharides via ABC routes to enter the cytoplsmic membrane of bacteria and into the inner thallus in an active transport way^33^. The metabolic preference of oligosaccharide by *L. delbrueckii* ssp. *bulgaricus* may vary with different types of bacteria. Often, thalli contain glucosidases such as α-glucosidases and β-fructofuranosidases^34^; thus, it has a relatively good ability to metabolize glucopyranosidic bonds and fructofuranose bonds in FOS, LOS3-1, and LOS4, which is consistent with the experimental results of fermentation. FOS with a DP of 7 is typically preferred for metabolism^35^, and FOS shows a better propagation effect than LOS3-1, indicating that fructofuranose bonds can be enzymatically cleaved more easily than glucopyranosidic bonds in thalli. The metabolism of LOS3-2 is relatively poor, thereby indicating that the end-product of LOS3-2 may be fructofuranose, which has a primary carbohydrate structure of a mannose bond, and its content in the thallus is low or the utilization rate of LOS3-2 is not high due to its weak activity in metabolism. Surprisingly, LOS4 showed better propagation ability and faster metabolic rate than FOS and LOS3-1. Except for glycosidase, LOS4 was rapidly transported inside the bacteria for direct metabolism due to its high affinity to the transmembrane protein or signaling enzyme.

During growth, *L. delbrueckii* ssp. *bulgaricus* utilizes different carbon sources to produce acetic acid and lactic acid (every 5 mL fermentation medium) via metabolism (Table 4). With the prolonging the fermentation period, the substrate where LOS4 is the carbon source showed an increase in the levels of acetic acid and lactic acid, which were produced during the metabolism of *L. delbrueckii* ssp. *bulgaricus (p* < 0.05). In other substrates that use Glc and FOS as carbon source, the amount of acetic acid and lactic acid produced during a time period of 24 h did not significantly differ from that produced for 10 h (*p* < 0.05). When LOS3-1 and LOS3-2 were used as carbon sources, the influence of fermentation time on the amount of acetic acid and lactic acid were not significant (*p* > 0.05). *L. delbrueckii* ssp. *bulgaricus* first utilizes glucose to foster propagation and lactic acid metabolism, followed by the use of oligosaccharides from lotus seeds. However, once glucose enters human body, it is often directly adsorbed by the human body or by other intestinal bacteria, and thus these cannot be utilized by probiotics in the human gastrointestinal tract. *L. delbrueckii* ssp. *bulgaricus* metabolizes glucose into lactic acid primarily via a glycolysis process for similar carbohydrates. The lactic acid ratio produced in the products was relatively high, and the ratio of lactic acid produced by the metabolism of oligosaccharide thallus was also relatively high, thereby indicating that metabolism of oligosaccharides by thallus involved mixed acid fermentation, and the specific fermentation mode relies on the substrate, the starvation of thallus, and the metabolic process^36^. Moreover, at 10 h after metabolism of LOS4 by *L. delbrueckii* ssp. *bulgaricus*, the number of thallus was larger than that of FOS. However, the amount of acetic acid produced was relatively low, which may be attributable to the early-stage of lactic acid glycolysis prior to the metabolism of LOS4 by bacteria using the mixed acid fermentation mode.

## Conclusions

In the present study, the MPLC-ELSD-DAD technique was applied to conveniently, automatically, and effectively separate and purify micro-molecular carbohydrates in large-scale (i.e., LOS2, LOS3-1, LOS3-2, and LOS4) from an aqueous extract of lotus seeds. LOS3-1 and LOS3-2 are isomerides, which show separation results that cannot be obtained by typical carbohydrate preparation and separation methods. Furthermore, this method allows the qualitative and quantitative analysis of these products, and provides references to the separation of other low-activity oligosaccharides in plants. The oligosaccharide structures in lotus seeds are not readily and directly adsorbed by the human body, and each separated component of specific structures shows different multiplication capacities, thereby demonstrating a certain structure-function relationship. The information generated in this study provides a novel approach for the directional adjustment and control of immunity where microorganisms in the human gut are targets for treatment of various diseases.

## Materials and Methods

### Extraction of Lotus Seed Oligosaccharides

Flash-frozen fresh lotus seeds (Green Acres Food Co., Ltd, Fujian, China) were thawed for 1 h, cored, and then dried in an air circulatory tray (DHG-9030, Tayasaf, Beijing, China). Lotus seeds were dried at 50 °C to retain only about 7% of its original water content. The desiccated lotus seeds were crushed with a mill (FW-80, Taisite Co., Tianjin, China), sifted through 60 meshes (pore size: 0.3 mm), and stored as experimental raw material. Dried lotus seed powder and deionized water were mixed at a liquid-solid ratio of 5 (v/w). Guo *et al*.^37^ described a method for the separation of starch from lotus seeds that prevented the degradation of oligosaccharides^38^. Then, the starch-depleted test sample solution was added to a back flowing water bath tank (HH-4, Changzhou Guohua Electric Co., Ltd, Jintan, Jiangsu, China) at 90 °C and a liquid-solid ratio of 70 (v/w). Upon completion of the reaction, the extracted solution was vacuum aspirated, and the residue discarded. The filtrate was speed vacuum-concentrated (BUCHI 409, Buchi Corp., New Castle, DE, USA) and 95% ethanol was added to three volumes of the test liquid, followed by overnight incubation at 4 °C and centrifugation (L530, Xiang Yi Centrifuge Instrument Co., Ltd. Changsha, China) The supernatant was freeze-dried (Model FD-4C-80, Beijing Fuyikang Instrument Company, Beijing, China) to powder consistency for subsequent processes.

### Optimization of the Separation Conditions for MPLC

The separation of oligosaccharides from lotus seeds was performed using a MPLC system (Interchim PuriFlash 450; Montluçon, France), which consisted of a MPLC bump, a DAD scanner (190–840 nm), a FLASH ELSD, a fraction collector, and a sample loading module (injection volume: 5 mL). All the system controls and data collection were fulfilled using the Interchim Software 5.0, and the MPLC column was constructed of specialty glass (36 × 460 mm, maximum operating pressure: 35 bar). The packing material was a Claricep Flash HILIC (20–35 μm, 100 A, 320 m^2^/g, Bonna-Agela Technologies, Tianjin, China), which was injected via a dry column-packing method. The elution solvent was an acetonitrile-water system. The wavelength range of the DAD scanner was 190–840 nm, and the following parameters of the ELSD were used: the nitrogen pressure was 3.5 bar, the drift tube temperature was 85 °C, and the gain value was 8. When optimizing the process, a certain amount of oligosaccharide powders extracted from lotus seeds using the above steps were dissolved in deionized water to prepare a solution with a certain concentration. After filtering through a 0.22-μm membrane (Millipore Corporation, Bedford, MA, USA), the solution was injected into an MPLC for separation and purification. The preparative chromatographic conditions were as follows: the mobile phase underwent isocratic elution or gradient elution, the column temperature was 25 °C, and the injection volume was 1 mL. Each time before a sample was injected into a chromatographic column, the column was equilibrated with three column volumes of the mobile phase (initial gradient). Furthermore, each time separation and purification was completed, a column volume of the mobile phase with the original gradient was used to wash the column. The flow rates of the mobile phase were adjusted to 30 mL, 45 mL, and 60 mL, respectively. Either isocratic elution (85% acetonitrile) or gradient elution was applied. Two sample concentrations of oligosaccharide extract were selected, namely, 0.2 g/mL and 0.4 g/mL. The influence of flow rate, gradient, and sample concentration on the separation and purification efficiency were studied, thus determining the optimized separation conditions.

### Purity Analysis of the Separated Products

A solution with different oligosaccharides solution obtained under optimal separation conditions was selected, which was evaporated in a rotary evaporator under a decompressed pressure at 65 °C to dry out the organic solvents, and then dried and frozen to obtain the compound powders for different oligosaccharides extracted from lotus seeds. The powders were dissolved in de-ionized water for HPLC analysis. A 1-mL aliquot was filtered through 0.22-μm membrane (Millipore Corporation, Bedford, MA, USA) and infused into an Agilent 1200 series rapid resolution LC system (Agilent Technologies, Palo Alto, CA, USA) at a 2 mg/mL concentration. The chromatography parameters comprised of a 20-μL sample volume, Agilent Hi-plex Na(Octo) column (300 × 7.7 mm, filler grain size: 8 μm) chromatographic column, distilled water flow phase, 0.6 mL/min flow rate, and 85 °C column temperature.

### LC/Q-TOF-MS Analysis

From a solution (2 mg/mL) containing different oligosaccharides extracted from lotus seeds, a 1-mL aliquot was filtered across a 0.22-μm membrane (Millipore Corporation, Bedford, MA, USA), and then injected into an LC/Q-TOF-MS system (1260/6520, Agilent Technologies, Santa Clara, CA, USA) to perform MS analysis. The following chromatographic conditions were used: the sample volume was 20 μL; the chromatographic column was produced by Agilent Hi-plex Na (Octo), (300 mm × 7.7 mm); the mobile phase was distilled water; the detector was a RID-10A differential detector; the flow rate was 0.6 mL/min; and the column temperature was 85 °C. The analytical conditions of Q-TOF-MS were as follows: cations were used for the ionization mode; the range of mass scanning was within m/z 50–1,000 Da; nitrogen was used as the drying gas; the gas temperature was 350 °C; the flow rate of the drying gas was 10 L/min; the pressure of the spraying gas was 40 psi; the voltage of capillary was 3.5 kV; the cracking voltage was 175 V; and the cone voltage was 65 V.

### GC-MS Analysis

Based on the method described by Lu *et al*.^4^.

### Evaluation of Bacteria-proliferating Effects of *L. delbrueckii* subsp. Bulgaricus

Based on the method described by Lu *et al*.^3^.

### Content Measurement of Short-chain Fatty Acid (SCFA) and Lactic Acid as Fermentation Products of *L. delbrueckii* subsp. Bulgaricus

To characterize samples, 1 mL of the bacteria fermentation broth was measured and poured into a 5-mL centrifuge tube. After the tube was placed in a water-ice bath for 10 min, 4 mL of sterilized and deionized water was added, and the solution was magnetically stirred and rotated for 2 min. The turbid solution was centrifuged at 5,000 *g* at 4 °C for 20 min, and the supernatant was again centrifuged. The supernatant after second round of centrifugation was collected and filtered using a 0.45-μm membrane, and poured into 1.5-mL gas samplers. Each sample was independently measured three times, and each time, the content of short-chain fatty acid in the fermentation broth was measured under the same conditions. The standard curve was slightly modified according to a method proposed by Feria-Gervasio *et al*.^39^, and standard methane acid, acetic acid, propanoic acid, butyric acid, sobutyric acid, and lactic acid solutions with five concentrations ranging from 5 to 25 mmol/L, as well as standard valeric acid and isovaleric acid solutions with five concentrations from 1 to 5 mmol/L, were prepared. The gas chromatographic method was used for quantitative analysis, and standard curves with the concentration being an x-axis (mmol/L) and the peak area being a y-axis were drawn to illustrate the results. The chromatographic conditions included using HP-INNOWAX chromatographic column (30 m × 0.320 mm × 0.25 μm) and an initial temperature of 100 °C maintained for 0.5 min, which was then increased to 200 °C at a heating rate of 4 °C/min. The whole process took around 20.3 min. The sample injection volume was 0.2 μL, the carrier gas was nitrogen whose flow rate was 20 mL/min; the flow rate of hydrogen as the fuel gas was 30 mL/min, the flow rate of the air as the oxidant gas was 300 mL/min, and the flow rate of make-up nitrogen was 19 mL/min; the temperature of the FID detector was 240 °C, and the temperature of the injection port was 240 °C. A splitless mode was applied.

The measurement of lactic acid content was primarily conducted using the HPLC method as described by Fernández *et al*.^40^. Approximately 20 mL of 4.5 mmol H_2_SO_4_ was added to 5 mL of the bacterial fermentation broth, which was mixed well, used for extraction for 1 h, and then centrifuged at 12,000 *g* for 5 min. Around 50 μL of the supernatant was isocratic eluted using a HPX-87H Aminex ion exchange column (300 × 7.8 mm, Bio-Rad, US), and the separation conditions were as follows: the flow rate was 0.7 mL/min, the column temperature was 65 °C, the mobile phase was H_2_SO_4_ (3 mmol/L), and the detector was an RID. An external standard method was used for quantification, and the standard curves obtained from the test are listed in Table 5.

### Statistical Analysis

The data were analyzed by one-way ANOVA using the DPS 7.50 system (Science Press, Beijing, China). Statistical significance was set at *p* < 0.05.

## Additional Information

**How to cite this article**: Lu, X. *et al*. Separation of Oligosaccharides from Lotus Seeds via Medium-pressure Liquid Chromatography Coupled with ELSD and DAD. *Sci. Rep.* **7**, 44174; doi: 10.1038/srep44174 (2017).

**Publisher's note:** Springer Nature remains neutral with regard to jurisdictional claims in published maps and institutional affiliations.

## Figures and Tables

**Figure 1:**
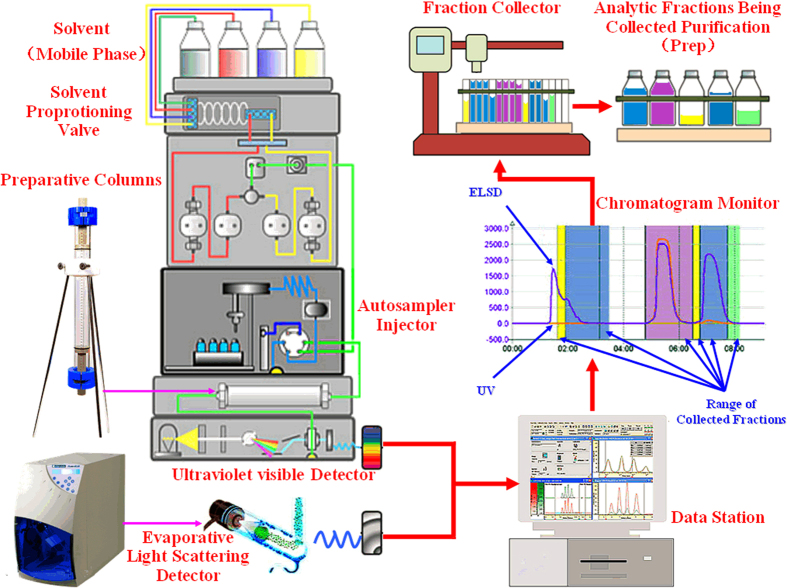
Schematic diagram of the HILIC-diode array detector (DAD)-ELSD system. It was drawn and taken by Dr. Xu Lu.

**Table 1:** The Q-TOF-MS analytical results of each oligosaccharide compound in lotus seeds.

Numbers	Mass fragments (m/z)	Adduct ion	Molecular formula	Matching degree
1	181.0716	[M − H]^−^	C_6_H_14_O_6_	99.60
2	365.1052	[M + Na]^+^	C_12_H_22_O_11_	99.68
	381.0472	[M + Ka]^+^		
3–1	527.1950	[M + Na]^+^	C_18_H_32_O_16_	99.52
	543.1257	[M + Ka]^+^		
3–2	527.1859	[M + Na]^+^	C_18_H_32_O_16_	99.51
	543.1328	[M + Ka]^+^		
4	689.2116	[M + Na]^+^	C_24_H_42_O_21_	99.35
	705.2048	[M + Ka]^+^		

**Table 2:** Linkage analysis of LOS2, LOS3-1, LOS3-2, and LOS4 isolated from lotus seeds.

Numbers	Methylation fragments	Connection method	Mass fragments(m/z)
1	2,5-Anhydro-1,3,4,6-tetra-O-methyl-D-mannitol	→2)-Fru*f*	41, 43, 45, 55, 59, 71, 75, 83, 85, 89, 99, 101, 111, 115, 125, 126, 143, 155, 156, 157, 175, 188, 221
2	2,5-Anhydro-1,3,4,6-tetra-O-methyl-D-glucitol	→2)-Fru*f*	41, 43, 45, 55, 59, 71, 75, 83, 87, 89, 99, 101, 111, 115, 125, 126, 143, 221
3	1,5-Di-O-acetyl-2,3,4,6-tetra-O-methyl-D-mannitol	Man*p*-(1→	43,45,87,101,117,129,145,161,205
4	1,5,6-tri-O-acetyl-2,3,4-tri-O-methyl-D-mannitol	→6)-Man*p*-(1→	43,87,99,101,113,117,129,161,173,189
5	1,5,6-tri-O-acetyl-2,3,4-tri-O-methyl-D-glucitol	→6)-Glc*p*-(1→	43, 87, 99, 101, 117, 129, 161, 189, 233
6	1,5-Di-O-acetyl-2,3,4,6-tetra-O-methyl-D-galactitol	Gal*p*-(1→	43,59,71,87,101,102,118,129,145,161,162,205
7	1,4,5-tri-O-acetyl-2,3,6-tri-O-methyl-D-glucitol	→4)-Glc*p*-(1→	43, 45, 87, 101, 117, 131, 161, 233

**Table 3:** Identification of the O-methylated alditol acetates derived from LOS2, LOS3-1, LOS3-2, and LOS4 isolated from lotus seeds.

Connection method	2	3–1	3–2	4
→2)-Fru*f* (mannitol)	—	17.335 min	—	17.346 min
→2)-Fru*f* (glucitol)	—	17.476 min	—	17.524 min
Man*p*-(1→	—	17.169 min	17.151 min	17.152 min
→6)-Man*p*-(1→	—	—	18.141 min	18.122 min
→6)-Glc*p*-(1→	—	22.163 min	22.179 min	22.155 min
Gal*p*-(1→	19.300 min	—	—	—
→4)-Glc*p*-(1→	23.412 min	—	—	—

**Table 4:** Concentration of short chain fatty acids (SCFAs) produced in the fermentation media with different carbon sources.

Carbon source	Time(h)	Acetic acid (mmol)	Lactic acid (mmol)
Control	10	2.001 (0.132)^fg^	11.916 (0.623)^b^
	24	1.999 (0.009)^fg^	12.081 (0.584)^b^
LOS3–1	10	2.429 (0.055)^ef^	38.183 (1.498)^e^
	24	2.468 (0.055)^ef^	37.633 (1.282)^ef^
LOS3–2	10	1.687 (0.099)^g^	28.179 (1.016)^g^
	24	1.877 (0.094)^fg^	30.193 (1.282)^fg^
LOS4	10	3.015 (0.028)^de^	53.903 (0.247)^d^
	24	3.431 (0.193)^cd^	70.971 (3.480)^c^
Glc	10	11.280 (0.757)^a^	155.711 (4.565)^b^
	24	11.392 (0.558)^a^	172.709 (9.804)^a^
FOS	10	4.051 (0.120)^bc^	30.083 (1.072 )^g^
	24	4.672 (0.009)^b^	44.040 (2.073 )^e^

*Results are expressed as mean standard deviation; values represent the average of triplicate analyses from three runs (n = 3). Lower case letters within the same column are significantly different (*p* < 0.05).

**Table 5:** Standard curve equations of SCFAs.

Short-chain fatty acids	Retention time (min)	Standard curve function	R^2^
Acetic acid	8.986 ± 0.075	*Y* = 62.415*x*-86.667	0.999
Propionic acid	9.986 ± 0.066	*Y* = 119.533*x*-42.163	0.999
Lactate acid	13.042 ± 0.006	*Y* = 83452.34*x* + 1579.27	0.999
Isobutyrate acid	10.271 ± 0.083	*Y* = 151.972*x*-21.665	0.999
Butyric acid	11.087 ± 0.047	*Y* = 180.075*x*-64.895	0.999
Isovalerate acid	11.544 ± 0.029	*Y* = 203.217*x*-26.577	0.999
Pentanoic acid	12.414 ± 0.020	*Y* = 185.125*x*-17.403	0.999

*x* is the concentration of fatty acid, mmol/L; *Y* is the peak area in the spectrum.
